# The Successful Use of Infliximab in a Relapsing Case of Susac's Syndrome

**DOI:** 10.1155/2020/9317232

**Published:** 2020-06-10

**Authors:** Suran L. Fernando, Therese Boyle, Annika Smith, John D. E. Parratt

**Affiliations:** ^1^Immunology Laboratory, New South Wales Health Pathology, Royal North Hospital, Sydney, Australia; ^2^Department of Clinical Immunology and Allergy, Royal North Hospital, Sydney, Australia; ^3^Sydney Medical School, University of Sydney, Sydney, Australia; ^4^Department of Neurology, Royal North Hospital, Sydney, Australia

## Abstract

Susac's syndrome is a rare and debilitating disease characterized by the triad of encephalopathy, branch retinal artery occlusions, and sensorineural hearing loss. All manifestations may not be clinically apparent at presentation resulting in delayed diagnosis. Early recognition of the syndrome may prevent disease sequelae such as permanent cognitive, visual, and hearing loss. We present such a case of Susac's syndrome that was also refractory to conventionally prescribed combination of immunosuppressive treatments including high-dose potent corticosteroids, intravenous cyclophosphamide, methotrexate, plasma exchange, rituximab, and mycophenolate. His disease was stabilized with infliximab in combination with a tapering course of low-dose prednisone. After 2 years of remission with TNF treatment, consideration is being given to ceasing therapy. He has the sequelae of bilateral sensorineural hearing loss but no visual impairment or cognitive deficits on follow-up with neuropsychometric testing. This is the first case report to our knowledge of the successful use of infliximab for a patient with Susac's syndrome that was necessary following treatment with cyclophosphamide and then rituximab.

## 1. Introduction

Susac's syndrome is a rare and potentially debilitating disease affecting small cerebral and retinal arteries and the cochlea, resulting in the triad of encephalopathy, branch retinal artery occlusions, and sensorineural hearing loss [1]. The pathogenesis of the disease is not fully understood but is likely to be a cytotoxic CD8+ cell-mediated endotheliopathy with consequent microinfarctions [2]. A potent immunosuppressive therapy is often required to reduce disease sequelae. We describe the effect of infliximab upon acute and chronic disease activity in this case of Susac's syndrome that was refractory to other immunosuppressive and immunomodulatory agents.

## 2. Case Presentation

A 30-year-old previously well man presented with a four-week history of progressive right-sided hearing loss, wide-based gait, and cognitive disturbance characterized by short-term memory loss, impaired attention span, and verbal fluency. The patient had neither a personal nor family history of pertinent medical problems, and he did not smoke, drink excessive alcohol, or take illicit drugs.

On examination, he had cerebellar signs characterized by dysdiadochokinesis and ataxia of gait.

**Figure 1:**
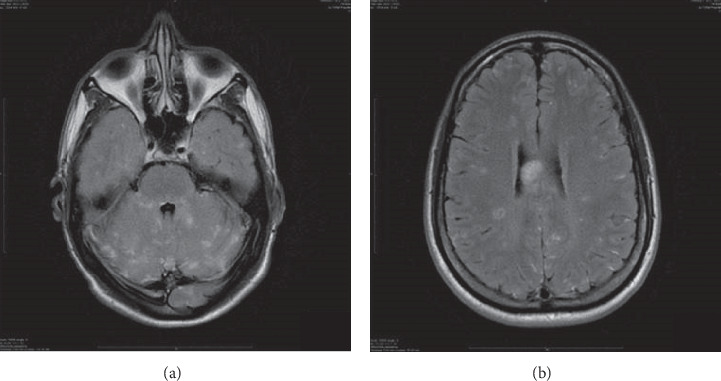
MRI brain (T2 axial section). Extensive postcontrast T2 FLAIR enhancement of periventricular and cerebellar diseases with patchy leptomeningeal enhancement accentuated by the FLAIR technique. (a) Large T2 FLAIR signal in the body of corpus (snowball lesions) and throughout the periventricular white matter. (b) Regions of T2 FLAIR signal correlated with DWI (b1000) diffusion restriction.

MRI of the brain at presentation demonstrated multiple hyperintense lesions in the corpus callosum, periventricular white matter, cerebellar hemispheres, and leptomeninges (Figure 1).

MRI of the spine was normal. The CSF showed a small pleocytosis with normal cytology and an elevated protein 3.06 g/L. There was an absence of oligoclonal bands in both CSF and serum. CSF microbiologic investigations revealed a Gram-negative stain, syphilis serology, and cryptococcal antigen. PCR analysis of the CSF did not detect EBV, CMV, HSV, VZV, enterovirus, *M. tuberculosis*, or fungal pathogens. Widespread triphasic waves consistent with encephalopathy but with no focal or generalised epileptic activity were demonstrable on an electroencephalogram (EEG). CT of the chest, abdomen, and pelvis did not reveal any lesions.

The full blood count, electrolytes, creatinine, and liver function tests were normal. The ANA, ANCA, anticardiolipin antibodies, and lupus anticoagulant were negative. Evaluation for autoimmune and paraneoplastic encephalitis with serum and CSF antibodies to NMDAR (*N*-methyl-D-aspartate receptor), AMPAR (*α*-amino-3-hydroxy-5-methyl-4-isoxazolepropionic acid receptor), LGI1 (leucine-rich glioma-inactivated 1), Caspr 2 (contactin-associated protein-like 2), GABA (gamma-aminobutyric acid) B receptor, and IgLON5 were negative, and ANNA-(antineuronal nuclear antibody-)1, ANNA-2, PCA (Purkinje cell antibody), PCA 2, Ma 1, Ma 2, CV2/CRMP5 (collapsin response mediator protein 5), Tr, and SOX antibodies were also not detected. Antiaquaporin-4 antibodies and anti-heat shock 70 antibodies were negative.

The differential diagnosis included acute disseminated encephalomyelitis, aquaporin-4 negative neuromyelitis optica [3], neurosarcoidosis, and primary angiitis of the CNS.

Despite treatment with intravenous methylprednisolone 1 g daily for 3 days followed by a tapering course commenced at 1 mg/kg daily, he suffered progressive bilateral hearing loss over the following four weeks and impaired vision from the right eye. Audiometry demonstrated low-frequency sensorineural hearing loss bilaterally. Neuropsychometric assessment revealed severe global cognitive impairment including difficulties with verbal fluency, as well as deficits in short-term memory and executive function.

A biopsy of the cerebellum and meninges was performed due to disease progression and lack of response to the high-dose corticosteroid therapy. The histopathology showed mild, nonspecific perivascular inflammation and a diffuse pial infiltrate dominated by CD8 T lymphocytes and macrophages with microinfarctions in the territory of the small pial arteries. There was no evidence of vasculitis or malignancy. Culture and PCR analysis of the specimen excluded mycobacterial or fungal infection.

**Figure 2:**
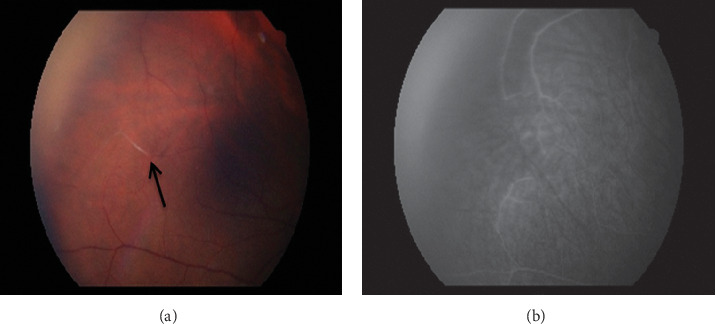
Fundal photography (right eye) showing a ghost vessel, likely secondary to previous branch retinal artery occlusion (arrow) (a). Late-phase fundal angiography with no perfusion noted in the corresponding territory (b).

The patient developed painless scotomata, and a branch retinal artery occlusion (BRAO) of the right temporal retina was identified on fundal photography and retinal fluorescein angiography (Figure 2). Subsequently, Susac's syndrome was diagnosed based on the triad of encephalopathy, right BRAO, and bilateral sensorineural hearing loss.

Methylprednisolone 1 g daily for 3 days was subsequently readministered followed by oral prednisolone 1 mg/kg daily concurrently with intravenous cyclophosphamide 1 g (15 mg/kg) monthly for 6 months and intravenous immunoglobulin (IVIg) 2 g/kg loading and 0.4 g/kg per month for 6 months. Adjunctive treatments included cotrimoxazole prophylaxis against *Pneumocystis jiroveci*, risedronate for osteoprotection, and aspirin to aid vessel patency.

Clinical remission was achieved by 6 weeks, but there was no improvement in the sensorineural deafness on the right side. Maintenance of remission was attempted with methotrexate 20 mg (0.3 mg/kg) weekly following cyclophosphamide.

Another clinical relapse, however, occurred 3 months after the maintenance therapy with methotrexate was started, characterized by recurrent bilateral hearing loss, bilateral BRAOs, and new T2 hyperintensities shown by MRI of the brain. Further remission was achieved with methylprednisolone 1 g daily for 3 days, and plasma exchange was administered daily for four consecutive days. In addition, rituximab 375 mg/m^2^ weekly for four weeks was used as an induction agent, and methotrexate was replaced with mycophenolate mofetil (MMF) 1.5 g BD. Prior to rituximab, enumeration of lymphocyte subsets on peripheral blood revealed 302 × 10^6^ CD19+ cells/L with CD19 suppression (<25 × 10^6^ cells/L) maintained for 18 months following therapy. Oral prednisolone was slowly tapered to a maintenance dose of 12.5 mg (0.18 mg/kg) daily.

Despite this treatment, the patient relapsed again two months later, i.e., 11 months following initial treatment, suffering a generalised tonic-clonic convulsion, encephalopathy, recurrent bilateral hearing loss, fresh BRAOs, and new MRI lesions. His neurological status improved after methylprednisolone 1 g daily was administered for 3 days. His dose of oral prednisolone was increased to 1 mg/kg daily, IVIg 2 g/kg monthly was recommenced, and MMF continued at a dose of 1.5 g BD. MRI brain with MR venogram demonstrated right transverse venous sinus thrombosis. A procoagulant screen was positive for the lupus anticoagulant. Review of a previous MR venogram 3 months prior showed that the thrombus had been present for some time and was not changing in morphology or size. For these reasons, aspirin rather than warfarin was continued as an anticoagulant.

**Figure 3:**
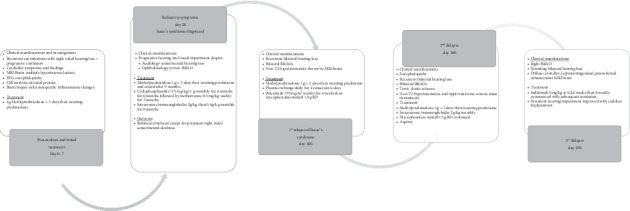
Timeline of case presentation with relapses and treatment moldalities.

A further clinical relapse occurred 3 months later, i.e., 14 months (Figure 3) after initial treatment was commenced, characterized by sudden painless partial visual loss in the right eye with new BRAO encroaching on the central retina, persisting hearing loss, and MRI changes demonstrating diffuse cerebellar, leptomeningeal, and parenchymal enhancement. Infliximab 5 mg/kg was started using a loading regimen at 0, 2, and 6 weeks for induction and then 8 weekly treatments for maintenance. Over the following 4 weeks, there was both clinical and radiological remission of disease. His dose of prednisone was tapered completely from 12.5 mg daily over 4 months. The thrombus resolved, and the patient returned to work. Two years after remaining on the maintenance infliximab therapy, the patient has been free of relapses, the visual acuity is normal, and there are no cognitive deficits on follow-up neuropsychological testing. He continues to be troubled by partial bilateral deafness, for which cochlear implantation has proven helpful. Consideration is being given to stepping down immunosuppression to MMF.

## 3. Discussion

Susac's syndrome is a very rare condition consisting of the clinical triad of encephalopathy, BRAO, and sensorineural deafness with an annual incidence of 0.024/100,000 (95% CI 0.010–0.047) [4]. It has a female preponderance with the peak age at onset occurring in the third and fourth decades of life [2]. A substantial proportion of patients including our patient did not present with the classical triad of manifestations, and therefore, diagnosis is often delayed, during which time disability may accrue [4]. The European Susac Consortium (EuSaC) proposed criteria for a definitive diagnosis characterized by the presence of the clinical triad, which was present in 56% of 192 patients, and probable Susac comprising 2 of the 3 clinical features in the remainder of the cohort [5]. This will allow for heightened suspicion for diagnosing, monitoring, and investigating undeclared or clinically subtle or silent manifestations enabling effective therapies to be implemented without delay.

The aetiology and pathogenesis of Susac's syndrome are unclear. The disease is proposed to be an autoimmune endotheliopathy characterized by cerebral microinfarction, hyalinised vessel walls due to collagen deposition, CD8+ lymphocytic inflammation, and perivascular lymphocytic cuffing in the leptomeninges, with enlarged and reactive endothelial cells leading to near occlusion of the vessel [6, 7]. The microvascular changes are indistinguishable from dermatomyositis [8]. Changes of vasculitis have been recently described in an autopsy case [9]. Antiendothelial cell antibodies have been identified, but whether they are pathogenic or epiphenomenon requires clarification [10, 11]. Recently, Gross et al. used immune profiling and phenotyping of blood and CSF samples with histopathologic studies of neural tissue from Susac's patients and patients with demyelinating diseases to reveal distinct mechanisms in these two neuroinflammatory conditions. Mouse models were also used to determine the effects of an antigen-specific cytotoxic CD8+ T cell (CTL) inflammatory effect against the CNS endothelium. They demonstrated that cytotoxic CD8+ T (CTL) cells mediated the vascular CNS injury, and they identified CTL-mediated endotheliopathy as a major pathogenic process in the disease [2]. Amelioration of disease was achieved in the mouse model by blocking adhesion and trafficking of CTLs using an anti-*α*4 integrin monoclonal antibody [2].

Coagulation abnormalities might modify or play a role in the pathogenesis of the disease. Although a procoagulant state is not consistently reported, protein S deficiency and factor V Leiden mutations have been described in case reports [12, 13]. Anticardiolipin antibodies and lupus anticoagulant have also been found in patients with Susac's syndrome [14, 15], but it is uncertain whether these are pathogenic. This is the first case of cerebral vein thrombosis described in Susac's syndrome suggesting that macrovascular as well as microvascular thrombosis can occur.

The optimal treatment for Susac's syndrome is not established, particularly in refractory or relapsing disease, due to the rarity of this condition. Therapeutic recommendations were initially largely based on anecdotal reports, case series, and pathological similarities with dermatomyositis [16]. In an early case series and review of the literature, most patients respond to oral prednisone (91% of 22 patients), IV methylprednisolone (87% of 8 patients), or IV cyclophosphamide (92% of 11 patient) although one patient who received cyclophosphamide developed hemorrhagic cystitis [17]. Hence, initial recommendations were for IV or the oral corticosteroid therapy and IV cyclophosphamide for refractory cases. IVIg was then introduced in the treatment regimen (usually 2 g/kg initially with the option of further doses at 0.4 g/kg monthly for a total of 6 months) in combination with either corticosteroids and/or adjuvant immunosuppression, e.g., azathioprine, mycophenolate, or cyclophosphamide [18–20]. Plasma exchange may be useful as an alternative to IVIg in combination with the adjunctive therapy, as was shown in eight patients who received between 3 and 10 exchanges, with six patients showing improvement and two demonstrating stabilization [21].

The biologic therapy was initially used in patients with Susac's disease refractory to conventional immunosuppression and immunomodulation in the mid-2000s due to its early success in patients with recalcitrant dermatomyositis [16] and especially in patients with sight-threatening BRAO [18]. Rituximab is frequently an effective therapy in immune-mediated diseases by removing the precursors of autoantibody-producing plasma cells and inhibiting autoantigen presentation to CD4 T-cells, perhaps in this case by abrogating the pathogenic effect of antiendothelial cell antibodies [2]. However, our patient relapsed following the rituximab therapy. The lack of efficacy of rituximab in our patient suggests that the pathogenesis of Susac's syndrome may not be mediated by autoantibodies alone. The successful induction of remission with cyclophosphamide suggests that potent inhibition of cell-mediated inflammation is required in severe and recalcitrant cases such as our patient.

Infliximab is a chimeric monoclonal antibody that binds to human tumor necrosis factor (TNF), thereby rapidly blocking with theendogenous TNF activity. TNF induces the production of proinflammatory cytokines (interleukins) by macrophages and endothelial cells, enhances leukocyte migration to sites of inflammation, activates neutrophils and eosinophils at inflammatory sites, and enhances production of acute phase reactants. Hence, the anti-TNF therapy has the advantage of disabling a final common pathway of inflammation irrespective of the initiating process. The effect of infliximab is relatively rapid as TNF is a preformed cytokine contained in the cytoplasm [22]. Furthermore, infliximab has been shown in diseases such as inflammatory bowel disease to induce apoptosis of T lymphocytes and enhance T regulatory cell function [23].

Infliximab was first successfully utilised in Susac's syndrome in 2011, in which a single dose was administered in a patient who was not responding to prednisone. The patient's symptoms of headache and ataxia improved within 24 hours [24]. However, follow-up treatment consisted of IVIG 2 g/kg administered 24 hours following clinical improvement, as well the commencement of cyclophosphamide 1 g monthly for 5 months. A multicentre retrospective case series demonstrated durable disease remission of ocular disease in 5 cases of Susac's syndrome with the TNF inhibitor therapy refractory to, on average, 3.2 immunosuppressive agents including prednisone, IVIg, mycophenolate, cyclophosphamide, azathioprine, methotrexate, and cyclosporine but not rituximab [25]. Three patients were treated with infliximab and two with adalimumab for a mean period of 2.86 years resulting in a reduction in the number of ocular relapses from 5.54 to <0.35 per year of follow-up during therapy. Studies in the prebiologic era revealed that immunosuppression had a minimal effect on the development of sensorineural deafness [26]. Further studies are required to determine whether biologic therapies given earlier for refractory disease improve auditory outcomes in Susac's patients. The finding of blocking T cell adhesion by anti-*α*4 integrin-intervention through the use of natalizumab in 4 patients in the study by Gross et al. for patients with progressive relapsing disease was encouraging despite the relapse in 2 patients once the therapy was ceased [2].

This is the first case to our knowledge of the successful use of infliximab for a patient with Susac's syndrome that was necessary following treatment with cyclophosphamide and then rituximab. It must be stated, however, that in a case series comprising three patients with Susac's syndrome successfully treated with infliximab, one was previously treated with rituximab. However, none of these patients had the cyclophosphamide therapy, and the precise time course between the administration of rituximab and infliximab was not presented in detail in this report [7]. It is also unknown as to when to cease the infliximab therapy. The natural history of Susac's syndrome is still unclear, but the disease seems to follow 3 different patterns: monocyclic (remission within 1-2 years without recurrence), polycyclic (recurrences over a period of greater than 2 years alternating with periods of remission), or chronic (prolonged, continuous course for more than 2 years) [27].

In conclusion, prompt and aggressive treatment of Susac's syndrome is recommended to improve prognosis [21]. The anti-TNF therapy may be an effective induction and maintenance therapy in refractory cases of Susac's syndrome. Further studies including elucidation of the pathogenesis are required to determine whether it should be incorporated earlier into therapeutic strategies in order to improve long-term outcomes. Consideration should also be given to its addition as a therapeutic option for refractory disease in the treatment algorithm and guidelines in Susac's syndrome [28].

## Data Availability

The data used to support the findings of this study are included within the article.
